# Femtosecond-resolved imaging of a single-particle phase transition in energy-filtered ultrafast electron microscopy

**DOI:** 10.1126/sciadv.add5375

**Published:** 2023-01-27

**Authors:** Ye-Jin Kim, Hak-Won Nho, Shaozheng Ji, Hyejin Lee, Hyunhyub Ko, Jonas Weissenrieder, Oh-Hoon Kwon

**Affiliations:** ^1^ Department of Chemistry, College of Natural Sciences, Ulsan National Institute of Science and Technology (UNIST), 50 UNIST-gil, Ulsan 44919, Republic of Korea.; ^2^ Center for Soft and Living Matter, Institute for Basic Science, 50 UNIST-gil, Ulsan 44919, Republic of Korea.; ^3^ Materials and Nano Physics, School of Engineering Sciences, KTH Royal Institute of Technology, Stockholm SE-100 44, Sweden.; ^4^ School of Energy and Chemical Engineering, UNIST, 50 UNIST-gil, Ulsan 44919, Republic of Korea.

## Abstract

Using an energy filter in transmission electron microscopy has enabled elemental mapping at the atomic scale and improved the precision of structural determination by gating inelastic and elastic imaging electrons, respectively. Here, we use an energy filter in ultrafast electron microscopy to enhance the temporal resolution toward the domain of atomic motion. Visualizing transient structures with femtosecond temporal precision was achieved by selecting imaging electrons in a narrow energy distribution from dense chirped photoelectron packets with broad longitudinal momentum distributions and thus typically exhibiting picosecond durations. In this study, the heterogeneous ultrafast phase transitions of vanadium dioxide (VO_2_) nanoparticles, a representative strongly correlated system, were filmed and attributed to the emergence of a transient, low-symmetry metallic phase caused by different local strains. Our approach enables electron microscopy to access the time scale of elementary nuclear motion to visualize the onset of the structural dynamics of matter at the nanoscale.

## Teaser

Energy-filtered ultrafast electron microscopy visualizes heterogeneous dynamics of VO_2_ nanoparticles with femtosecond precision.

## INTRODUCTION

Visualizing structural rearrangements at the atomic/molecular level is essential in understanding the functions of matter. Once isolated, the intermediate and transitional structures of a molecule during a chemical reaction may be routinely determined via ultrafast spectroscopy with femtosecond temporal precision (*1*–*4*). When the structural degrees of freedom increase, e.g., condensed matter comprising countless atoms or organized molecular units, local nanoscale structural defects become prevalent, and thus, the physical processes of each singularity do not proceed as those in the bulk and even diverge. These structural defects are limiting factors of device performance, and, if controlled, they may yield functional benefits and be exploited to overcome the current technological challenges in fields ranging from catalysis (*5*, *6*) to quantum computing (*7*, *8*). Because the ensemble natures of spectroscopic measurements do not reveal the characteristic transition of each nanoscopic structure, simultaneous spatiotemporal imaging is required to resolve complex processes, hierarchically spanning small-amplitude, ultrafast atomic displacements to collective structural rearrangements at expanded spatiotemporal scales (*9*–*15*). Using pulsed electrons in a transmission electron microscope (TEM), ultrafast electron microscopy (UEM) enables time-resolved imaging with a spatial resolution approaching (sub)nanometer precision (*14*). The temporal resolution has been governed by the duration of the imaging electron pulse at the moment of transmitting a specimen.

For imaging electron pulses, the duration is mainly determined at the initial stages of photoemission (*16*–*20*). The mismatch of photon energy and the work function of the photocathode, inhomogeneities on the surface, and bandwidths of the photoemission-driving pulses contribute to a shot-to-shot variation in electron energy. This energy spread of electron pulses develops a chirp, which is an energy (*E*)–time (*t*) correlation defined as a phase-space slope, resulting in the temporal broadening of the pulses because the leading electrons with higher energies accelerate and those with lower energies are retarded during propagation (*21*–*23*).

Several schemes have been proposed to circumvent the pulse broadening. Under electrostatic acceleration, a femtosecond resolution requires an extremely short distance between the source of photoelectrons and a sample (*24*, *25*). Minimization of the excess energy above that of the work function aids in suppressing the broadening (*26*). A radiofrequency electric field may decelerate the leading electrons and accelerate the trailing ones, compressing the pulse at a sample plane (*27*, *28*). The durations of single-electron pulses may reach 10 fs with a microwave cavity and even attoseconds under a terahertz optical field (*27*, *29*, *30*). Recently, femtosecond-duration pulses were produced by chopping a continuous electron beam (*31*). However, these single-electron–based approaches, with low electron counts, compromise the signal-to-noise ratio. For practical use, numerous electrons (typically >10^7^) should be collected at each time frame to yield a satisfactory signal-to-noise ratio. Considering electron loss during propagation, the Poisson distribution of a single electron per pulse, and an acceptable integration time (<100 s), imaging should be repeated at >1 MHz, at which the reversibilities of dynamic phenomena are rarely satisfied, limiting the scope of UEM. The detrimental repetition issue may be overcome if each pulse contains multiple electrons at a lower repetition rate. In dense electron packets, however, the total electron-energy spread and the pulse broadening are mainly caused by Coulomb repulsion among electrons.

**Fig. 1.**
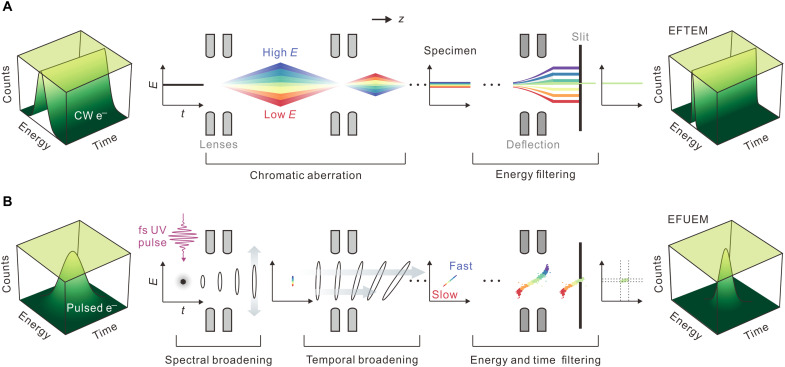
Concept of EFUEM. Electron distribution is defined in the energy-time phase space (left column). (**A**) Correction of a spatial chromatic aberration. For thermionic/field-assisted continuous wave (CW) electrons, the chromatic aberration, which is generated by the electron energy distribution and prespecimen electromagnetic lens systems, degrades the spatial resolution. The energy gradients of electrons are angularly dispersed through the magnetic prism in an electron-energy-loss spectrometer (“deflection”). The exit slit (energy filter) of the spectrometer gates the energy range of interest. The resultant three-dimensional correlation map shows the narrowed energy distribution, which minimizes the chromatic aberration for an enhanced spatial resolution. (**B**) Correction of a temporal chromatic aberration. The concept of EFUEM is benchmarked from that of EFTEM in (A) but with a timed photoelectron bunch instead. The electrons experience space-charge forces during their propagation at two steps: spectral dispersion at the first stage of photoemission and longitudinal (temporal) dispersion until the bunch reaches the specimen—these forces deteriorate the instrument response function. Unlike a continuous wave, pulsed photoelectrons subjected to Coulomb repulsion develop a well-established energy-time correlation, chirp. Temporal accuracy on a femtosecond (fs) scale may be achieved using picosecond-duration chirped pulses when the bunch is gated with a narrow energy range. UV, ultraviolet.

In this study, we demonstrate energy-filtered UEM (EFUEM), which exhibits a femtosecond resolution with a conventional energy filter for use in a TEM without pulse compression. As energy-filtered TEM (EFTEM) is advantageous for use in enhancing image contrast/resolution by mitigating chromatic aberration effects, which blur images (Fig. 1A) (*32*, *33*), the temporal resolution is controlled in EFUEM by selecting chirped photoelectrons of distinct kinetic energies using the postspecimen energy filter (Fig. 1B). A multiphotoelectron packet is generated, with the space-charge force critical in developing the chirp. By gating the detection range of electron energy, the frame time of images or diffraction patterns is controllable, providing data regarding the structure in real or reciprocal space, respectively. The temporal resolution is then determined by the slit width of the energy filter instead of the duration of the electron pulse. The concept enables the resolution of ultrafast structural responses in matter beneath a temporally broad photoelectron bunch without delicate instrumental modification or compromising the large number of electrons, as in the single-electron approach. The concept of gating chirped photoelectrons was proposed by Baum and Zewail (*22*) a decade ago and was recently demonstrated in a simulation of ultrafast electron diffraction (*34*). In our study, we demonstrate ultrafast real-space femtosecond-resolved imaging using EFUEM, observing the phase-transition dynamics of vanadium dioxide (VO_2_) to study its heterogeneous nature with different strains exerted on individual nanoparticles (NPs) in an ensemble.

## RESULTS

### Chirped photoelectrons in UEM

To map the *E*-*t* phase space of chirped photoelectrons at the specimen plane, we used photon-induced near-field electron microscopy (PINEM), wherein a nanostructure (a network of Ag nanowires) was excited with an optical pulse to form a near field, which couples evanescent photons to free electrons (*35*). As a result, the electrons gained or lost energy by multiple quanta of the photon energy when the electron and photon pulses overlapped spatiotemporally. Following the time-dependent changes of the zero-loss peak and populated gain/loss peaks, the relationship between *E* and *t* of the chirped photoelectrons could be obtained.

**Fig. 2.**
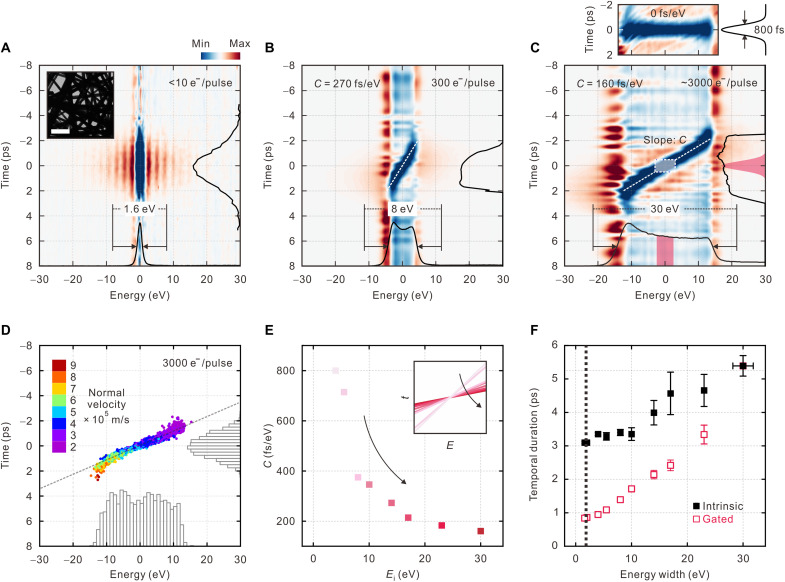
Chirp dependence on *E*_i_. (**A** to **C**) *E*-*t* phase-space maps of photoelectrons with various electron energies from 1.6 to 8 and 30 eV, respectively, depending on the number of electrons per pulse (*N*_e_). Integrated temporal and energy profiles are shown along each axis. Inset in (A) shows a bright-field TEM image of Ag nanowires. Scale bar, 500 nm. The *C* values and numbers of electrons per pulse are denoted within each panel. The lower limit of the pulse duration of the 30-eV photoelectrons is 840 ± 30 fs based on the residual spread around the linear fit of the chirped *E*-*t* profile with slope *C*; see the top. (**D**) Simulated *E*-*t* phase-space map for a photoelectron bunch (*N*_e_ = 3000) in a relative time domain at the sample plane. Color level denotes the initial velocities of the photoelectrons immediately after photoemission. Integrated temporal and energy distributions of the photoelectrons are shown along each axis. (**E**) Dependence of *C* on *E*_i_. Inset shows the slope of chirp correlation with the corresponding *E*_i_ (arrow). (**F**) Dependence of imaging pulse duration on *E*_i_ (intrinsic, closed squares) and slit width (*E*_g_, gated, open squares). A dotted line shows that energy gating can achieve a factor of 4 of improvement of the temporal resolution at the same energy width.

The time-resolved differential electron energy-loss (EEL) spectra shown in Fig. 2 (A to C) with respect to the reference spectrum measured at a negative time delay (Δ*t* = −8 ps) constitute the *E*-*t* phase-space maps characterizing the photoelectron packets. At a single electron per pulse regime, electron side bands with the energy gain/loss of photon quanta are observed without chirp (Fig. 2A). The intrinsic energy spread (*E*_i_) is controlled by varying the ultraviolet laser fluence at the photocathode from 10 to 500 μJ/cm^2^ to yield approximately 10 to 3000 photoelectrons per packet at the specimen plane. Figure 2 (B and C) clearly shows electron chirp, i.e., the decrease in electron energy with increasing electron arrival time. The apparent slope (δ*t*/δ*E*), defined as the chirp coefficient *C*, decreases as the energy spread increases. In a similar configuration under the instrumental conditions, chirped electron packet in the experiments with longitudinal velocity distribution of the electrons therein was simulated using the finite element method (Fig. 2D) (*19*). The slopes of the time-dependent shifts of zero-loss energy depletion in the differential maps of a series of energy distributions are fitted to obtain the *E*-*t* correlation represented by *C*. For the 8-eV packet containing 300 electrons (*t*_i_ = 4.0 ± 0.4 ps; *t*_i_ is the intrinsic duration), *C* is 270 fs/eV (Fig. 2B), whereas for 30-eV photoelectron pulses (*t*_i_ = 5.4 ± 0.3 ps) containing approximately 3000 electrons, *C* is 160 fs/eV (Fig. 2C). The chirp varies nonlinearly as a function of *E*_i_ (Fig. 2E), which indicates that a larger *E*_i_ (smaller *C*) yields an enhanced temporal resolution at a gating energy of the same width.

### Gating chirped photoelectrons with femtosecond durations

After subtracting a linear fit from the chirp in the correlation map of the 30-eV packet, we obtained the temporal width of the energy dip to be 840 ± 30 fs (top in Fig. 2C), which corresponds to the optimal temporal cross-correlation (*t*_cc_) that is mainly governed by the optical pump used in this study (*t*_p_ = 550 ± 20 fs; *t*_p_ is the duration of the pump pulse). A larger temporal width than *t*_p_ arises from the convolution with the spectrometer resolution (dispersion). The overall temporal resolution [instrument response function (IRF)] in EFUEM is determined as

$$\mathrm{IRF}\approx \sqrt{t_{p}^{2}+t_{g}^{2}+t_{j}^{2}}\geq t_{\mathrm{cc}}$$ (1)

where *t*_g_ is the temporal width of the gated electrons, i.e., *t*_g_ = *C*∙*E*_g_; *E*_g_ is the slit width of the energy filter; and *t*_j_ is the temporal uncertainty related to the spectral jitter (*E*_j_) of the energy filter based on *t*_j_ = *C*∙*E*_j_. The velocity mismatch between the pump and electron pulses is ignored because the 200-keV electron and pump pulses are almost colinearly incident on thin (<100-nm) samples. At *C* = 160 fs/eV, *E*_j_ ≈ 1 eV, and the mechanical limit of *E*_g_ = 2 eV, the IRF approaches the temporal limit of *t*_cc_. *C* may be further reduced to 40 fs/eV by replacing the photocathode with a smaller one and/or changing the photoemission environment (fig. S1). The slit of the energy filter gates the photoelectron bunch at Δ*E* = 0 eV, while minimizing the contributions from nonlinear chirps, as shown in Fig. 2C. Postspecimen energy gating results in almost a factor of 4 of improvement of temporal resolution to that obtained by reducing *E*_i_ and thus by relaxing the space charge (Fig. 2F). When *E*_i_ is reduced from 30 to 1.6 eV, *t*_i_ is improved from 5.4 ± 0.3 to 3.1 ± 0.1 ps. In contrast, gating the 30-eV photoelectron packet with a slit width of 2 eV would yield a *t*_g_ of 860 ± 80 fs (fig. S2). Energy gating is also favorable in terms of electron currents, which is related to the integration time for imaging. For the same temporal width (*t*_i_ = *t*_g_) at the limit of the comparison (*t*_i_ ≈ 3 ps), the current of the gated photoelectron pulse is approximately 40 times larger than that of the intrinsic pulse (fig. S3). The difference becomes smaller at longer temporal widths. Therefore, energy gating is advantageous in that a larger number of electrons may be used while maintaining the same temporal resolution as that of a single-electron pulse.

### Femtosecond-resolved imaging in EFUEM

A proof-of-concept study was performed using a polycrystalline VO_2_ film, which exhibits an insulator-to-metal transition (IMT) from a low-temperature semiconducting phase (bandgap ≈ 0.6 eV) with a low-symmetry monoclinic structure, *M*1, to a high-temperature metallic phase with a high-symmetry rutile structure, *R*, in the space groups *P*2_1_/*c* and *P*4_2_/*mnm*, respectively (*36*–*40*). Excitation using femtosecond optical pulses induces an ultrafast transition from the *M*1 to the *R* phase. Ultrafast diffraction and spectroscopic measurements revealed that light-induced IMT occurs within tens to hundreds of femtoseconds, followed by slow lattice reorganization at a time scale of up to hundreds of picoseconds (*41*–*47*). This low- to high-symmetry conversion is reflected in changes in the diffraction intensities of the VO_2_ lattice Bragg peaks, particularly the disappearance of lower-symmetry reflections due to dissociation of dimerized V pairs (*44*).

**Fig. 3.**
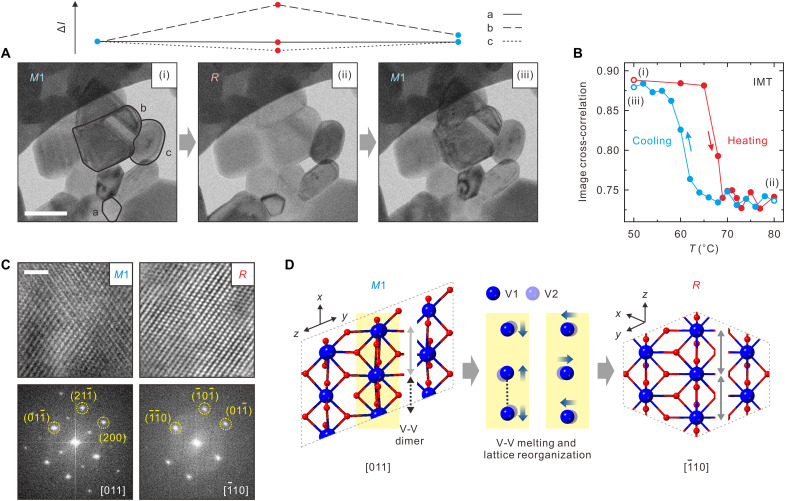
Phase transitions of VO_2_ NPs. (**A**) In situ bright-field TEM images of VO_2_ NPs captured during a heating and cooling cycle, which leads to the transition from the monoclinic (*M*1) to rutile (*R*) phase and then back to the *M*1 phase. The steady-state temperatures of stages (i), (ii), and (iii) are 50°, 80°, and 50°C, respectively. Scale bar, 200 nm. Profiles of the diffraction intensities within NPs a to c are shown above. (**B**) Hysteresis loop of the phase transition of the NP in (A) obtained from image cross-correlation. Heating and cooling cycles follow red (down) and blue (up) arrows, respectively. (**C**) High-resolution bright-field TEM images of NPs in two different phases (*M*1 and *R*) and their corresponding FFTs. Scale bar, 5 nm. Representative Miller indices are denoted in the bottom panels. (**D**) Crystal structures in the *M*1 and *R* phases. The two adjacent V atoms, V1 (top) and V2 (bottom), along the V-V direction in the *M*1 phase reorganize to dissociate the dimeric bond in the *R* phase.

In bright-field TEM, intraparticle diffraction contrast is extremely sensitive to crystallographic changes and strain fields, providing a direct measure of structural transformation (*13*–*15*, *48*). By introducing an objective aperture to select only the central electron beam at a back focal plane below the specimen, the characteristic diffraction contrasts of various VO_2_ NPs in the film are distinguished [(i) in Fig. 3A]. Depending on the zone axis of each NP, the contrasts initially appear dark (particle B) or bright (particle C). When the steady-state temperature of the specimen was raised from 23° to 68°C and above, the diffraction contrasts of most VO_2_ NPs changed [(ii) in Fig. 3A and movie S1]. The contrasts returned to the original state upon cooling to the initial temperature (iii). Cross-correlation of the diffraction contrasts in the NP b in (i) is tracked during the entire heating-cooling cycle, revealing a hysteresis loop in the transition between the *M*1 and *R* phases, as shown in Fig. 3B. High-resolution bright-field TEM images of the VO_2_ NPs in both phases and the corresponding fast Fourier transforms (FFTs) were used to identify the crystal structures at each phase and elucidate the origin of the change in diffraction contrast (Fig. 3C). In the *M*1 phase along the [011]_*M*1_ zone axis, lattice fringes caused by the V-V dimers are observed, as shown by the lower-symmetry reflections of the corresponding FFT. At elevated temperatures, the bonds are cleaved, resulting in the loss of the initial lower-symmetry reflections and transition to the *R* phase with half the unit cell of the *M*1 phase aligned to the [$\$\bar{1}\$$10]*_R_* zone axis. The rearrangement of the V atoms along and perpendicular to the V-V axis at elevated temperatures equalizes the two bonds, as shown schematically in Fig. 3D. This accounts for the changes in the diffraction contrasts of the NPs during the IMT. Accordingly, the changes in diffraction contrast in time-framed images may be used in tracking the light-induced ultrafast IMTs of VO_2_ NPs. The IRF may thus be stringently quantified if we follow the immediate changes in the diffraction contrasts upon photoexcitation of VO_2_ NPs, with symmetries that change within 300 fs upon photoexcitation (*42*, *43*, *45*, *47*).

**Fig. 4.**
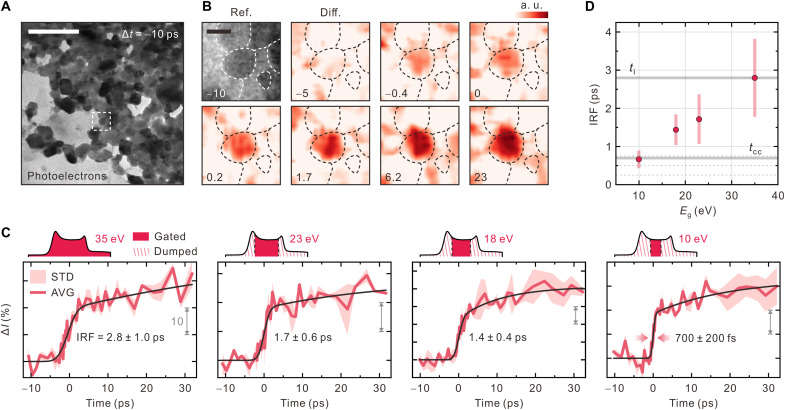
Femtosecond-resolved real-space imaging by gating chirped photoelectron bunch. (**A**) Bright-field image of an ensemble of VO_2_ NPs captured using photoelectrons with *E*_i_ = 35 eV before photoexcitation (Δ*t* = −10 ps). Scale bar, 500 nm. The NP of interest (P1) shown in (A) exhibits an immediate phase transition upon photoexcitation. (**B**) Time-resolved images of P1 obtained at *F* = 18 mJ/cm^2^. Corresponding Δ*t* values are shown in picoseconds in each panel. False-color images are a series of difference images over time referenced to Δ*t* = −10 ps. Red indicates higher electron counts. Peripheries of the NPs are outlined with dotted lines in each panel. Scale bar, 100 nm. a.u., arbitrary units. (**C**) Time-resolved intensity profiles of the central VO_2_ NP (P1) shown in (B) obtained using gated photoelectrons. The averaged transients (AVG, solid line) of three independent measurements are shown with their standard deviation (STD) values. Exponential fit curves are also shown (black solid lines). The slit width of the energy filter is shown in the corresponding spectrum above each panel. (**D**) Experimental IRFs obtained at different *E*_g_ values. *t*_i_ and *t*_cc_ are also shown.

Figure 4A shows a bright-field image captured using photoelectron pulses with an energy width of 35 eV at Δ*t* of −10 ps before photoexcitation and *C* = 100 fs/eV (fig. S4). The differential time-framed images referenced to the image of Δ*t* = −10 ps shown in Fig. 4B were extracted from movie S2. The NP of interest (P1) within the dashed box in Fig. 4A exhibits an instant change in diffraction contrast upon photoexcitation at a fluence (*F*) of 18 mJ/cm^2^ (Fig. 4B), which is well above the reported threshold (*F* ≈ 8 mJ/cm^2^) (*41*, *43*–*45*). To demonstrate the IRF approaching the time scale of the ultrafast IMT, time-resolved images were obtained by gating the photoelectrons using various slit widths (Fig. 4C). Without gating, the IRF of the 35-eV photoelectron pulse used in time-resolved imaging is 2.8 ± 1.0 ps, which is approximately half of the duration obtained from PINEM in Fig. 2F where *t*_i_ was 5.4 ± 0.3 ps. We infer that the determination of the duration of photoelectron pulses by PINEM can be overestimated because of the nonlinearity of electron-photon interaction (*23*, *49*, *50*). Because the initial structural response of VO_2_ to pulsed photoexcitation occurs on the time scale of ≤300 fs (*42*, *43*, *47*), the fastest rise time of the intensity profiles shown in Fig. 4D should correspond to the IRF. When the energy window is narrowed via gating, the rise time decreases. With *E*_g_ decreasing from 35 to 23, 18, and 10 eV, the IRF gradually improves to 700 ± 200 fs, which is reasonable considering the *t*_cc_ of approximately 800 fs. The IRF in time-resolved imaging is obtained to be shorter than that estimated from PINEM spectra (Fig. 2F) and considered to be more accurate in terms of measuring temporal width. This discrepancy is mainly attributed to the further development of the chirp through propagation after the specimen plane, where dense photoelectrons in a pulse can still exchange energy with each other as the pulse propagates longitudinally, resulting in additional energy spread (*51*).

### Revealing particle-dependent dynamics

**Fig. 5.**
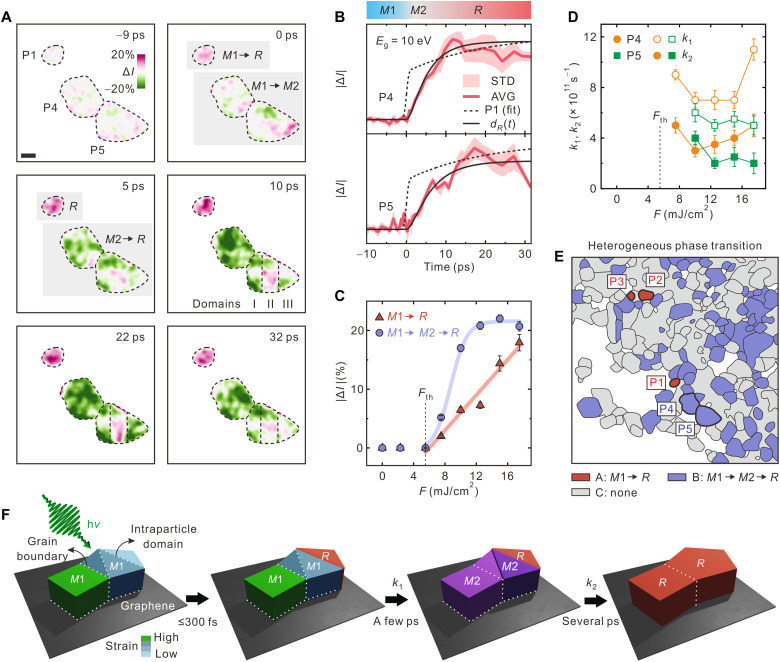
Single-particle phase transition. (**A**) False-color time-resolved images of P1, P4, and P5 shown in (E) referenced to the images at Δ*t* = −10 ps. Scale bar, 100 nm. Corresponding Δ*t* values are shown in each panel. Color scale indicates intensity change. (**B**) Time-resolved intensity profiles of P4 and P5 obtained at *E*_g_ = 10 eV. The intensity profile of P1 is shown as a dotted line for comparison. The delays in P4 and P5 are 0.8 ± 0.4 and 1.4 ± 0.7 ps, respectively. Biexponential fits for the formation of the *R* phase, as modeled according to Eq. 2, are shown as solid black lines. Phases *M*1, *M*2, and *R* are shown in their corresponding time windows. (**C**) Dependences of the diffraction intensity change for the two different IMTs on *F*. (**D**) Dependences of the rates in the consecutive transitions of P4 and P5 on *F*. (**E**) Map of the heterogeneous phase transition in a NP ensemble. Region A (red) contains NPs undergoing direct transition from *M*1 to *R*, whereas NPs in region B (purple) respond after a delay of a few picoseconds for the formation of the intermediate phase, *M*2. Intensity change is negligible in region C (gray). (**F**) Schematics of the ultrafast phase transition of VO_2_ NPs on graphene. For strain-free NPs, relatively free boundary conditions enable instant transition from the *M*1 to the *R* phase upon photoexcitation, while strained NPs transform to the transient *M*2 phase. Once the potential energy accumulating at the NP reaches its maximum, it drives the further transition to the *R* phase.

Unlike P1 shown in Fig. 4 and P2 and P3 shown in fig. S5, which exhibit instant changes upon photoexcitation (Δ*t* = 0), the structural responses of most NPs in the field of view are delayed by a few picoseconds. To elucidate the origin of the delay during the IMT, the structural dynamics of other NPs, e.g., P4 and P5 shown in Fig. 5A (extracted from movie S3), are compared to those of P1. The intensity changes in the diffraction contrasts of P4 and P5 commence at 0.8 ± 0.4 and 1.4 ± 0.7 ps after Δ*t* = 0, respectively, which become clearer when the photoelectrons are gated with *E*_g_ = 10 eV than when using those with *E*_g_ > 10 eV (Fig. 5B and fig. S6). These delays are attributable to the formation of a transient, monoclinic, Mott-insulating phase *M*2, because the *M*1 and *M*2 phases exhibit the same crystal symmetry, and thus, structural probing should be blind to the transition between these two phases (*52*, *53*). Hence, the following slow rise in several picoseconds corresponds to the structural change from the intermediate *M*2 phase to the final *R* phase. Under the mechanistic framework of a consecutive IMT (*M*1 → *M*2 → *R*; fig. S7), the time-dependent density of the *R* phase [*d_R_*(*t*)] is expressed as

$$d_{R}(t)=d_{M1}(0)\left[1+\frac{1}{k_{1}-k_{2}}(k_{2}e^{-k_{1}t}-k_{1}e^{-k_{2}t})\right]$$ (2)

where *d*_*M*1_(0), *k*_1_, and *k*_2_ represent the initial density of the photoexcited *M*1 phase and the rate constants of the first and the second step in the consecutive transitions, respectively. In this model, the delay corresponds to the induction time of the formation of the *R* phase, due to the preceding *M*1 → *M*2 transition with *k*_1_. Last, *k*_1_ and *k*_2_ are deduced to be (8.5 ± 0.7) × 10^11^ and (2.2 ± 0.6) × 10^11^ s^−1^ for P4 and (7.0 ± 0.7) × 10^11^ and (1.8 ± 0.5) × 10^11^ s^−1^ for P5, respectively (Fig. 5B).

While such delayed structural responses to photoexcitation are absent in strain-free, single-crystalline bulk samples (*42*, *44*, *47*), they are prevalent in polycrystalline samples (*41*, *45*). The polycrystalline VO_2_ NPs supported on the graphene substrate in this study are subjected to interfacial strain due to dipole-dipole interactions (*54*), leading to the emergence of the *M*2 phase even at room temperature for some particles (fig. S8), similar to the stabilization of the steady-state *M*2 phase when VO_2_ is formed on a hexagonal boron nitride flake (*55*). The intraparticle domains and grain boundaries of P4 and P5 (fig. S9), with larger grain sizes than those of P1, also favor the persistence of the transient *M*2 phase. For P5, which comprises three intraparticle domains, the change is locally different at each domain (I to III). Conversely, single-domain P1 is relatively free of interfacial strain, and thus, direct transition from the *M*1 to the *R* phase is favorable. Most of the NPs in the field of view are likely highly strained due to their crystal structures (fig. S10) and undergo the consecutive IMT via the *M*2 phase with an induction time of a few picoseconds, as shown in movie S4. We note that the slow rise component with <10% change in Fig. 4C during the first 10 ps following the ultrafast switch to the *R* phase may correspond to an additional thermal process by the latent heat released during the ultrafast IMT.

The changes in diffraction intensities in the two IMTs exhibit different *F* dependencies, with similar average thresholds of 5 mJ/cm^2^ (Fig. 5C and fig. S11). For NPs undergoing direct transition *M*1 → *R*, the *F* dependence is monotonic. Conversely, for strained NPs, the successive transition (*M*1 → *M*2 → *R*) is not linearly dependent on *F*, displaying a steeper dependence at low *F* values and saturation at >10 mJ/cm^2^. Our particle-selective observation is consistent with that of a previous ensemble study (*45*), based on which it was proposed that the IMT is limited to the *M*1 → *M*2 transition at lower *F* values, whereas a further transition to *R* is accessible at higher *F* values. The discrepancy arises from the fact that the ensemble measurements could not resolve a characteristic transition of each VO_2_ NP with a unique boundary condition. *k*_1_ and *k*_2_ are almost independent of *F* (Fig. 5D), indicating that the processes are barrierless. On the basis of our EFUEM study, the emergence of the *M*2 phase toward the *R* phase with the transient delay may indicate that Mott-like phase transitions are fully responsible for the IMTs of the strained VO_2_ NPs without concomitant Peierls transitions, even at well above the threshold *F*.

Figure 5E shows the overall heterogeneity of the phase-transition dynamics in VO_2_ NPs. The NPs in region A undergo the instantaneous phase transition from the *M*1 to the *R* phase, whereas the NPs in region B are strained and undergo the IMT via the intermediate *M*2 phase. The remaining NPs in region C display no substantial changes of diffraction contrasts, because of the tilt of zone axes, residual heat due to the stroboscopic nature of the measurements, and/or the presence of the *M*2 phase stabilized at the initial condition via strain (*56*–*59*). Several NPs in region C exhibit ultrafast IMT and slow lattice reorganization over a time scale of tens of picoseconds, as observed at different tilt angles (fig. S12).

## DISCUSSION

The duration of energy-filtered electron pulses could be controlled by selecting the bandwidth of energy gating. When gated by an energy width of 10 eV (*E*_i_ = 35 eV) to achieve a pulse duration of 700 fs, the number of useful electrons at a repetition rate of 50 kHz was approximately 10^7^ per second; thus, the total integration time per frame was 10 to 20 s. In our approach, the temporal resolution of ultrafast imaging was additionally limited by the degree and dispersion of the chirps of probing electron pulses, the spectral jitter of the energy filter, and the duration of the pumping optical pulses to initiate structural change. If these factors are improved, the temporal resolution of ultrafast imaging may reach <100 fs, which surpasses the time scale of serial atomic displacement. Using the energy-filtered photoelectrons, the ultrafast IMTs of VO_2_ NPs were visualized, revealing their unique behaviors associated with nanoscale strain. As shown in Fig. 5F, a NP under negligible strain, such as the bulk single-crystalline VO_2_, transforms from the initial *M*1 phase directly to the *R* phase upon photoexcitation, with a uniform change across the entire NP. When a NP in the *M*1 phase is partially strained, its metallic *M*2 phase emerges for the first few picoseconds, retaining the parent lattice structure, which then subsequently undergoes the structural transition to the *R* phase. Our observations yield insight into the nature of the phase-transition dynamics within strongly correlated electron systems toward fabricating ultrafast optoelectronic devices based on phase tuning via strain control at the nanoscale.

## MATERIALS AND METHODS

### Preparation of VO_2_ thin films

Polycrystalline VO_2_ films were prepared on graphene substrates via the sol-gel method (*60*, *61*), which consists of spin-coating and a subsequent annealing process under an Ar atmosphere. The coating solution was prepared by dissolving the precursor vanadyl acetylacetonate (99.99% purity; Sigma-Aldrich, St. Louis, MO, USA) in methanol and then spin-coated onto graphene substrates covering Cu TEM grids (two to six layers, Graphene Supermarket, Ronkonkoma, NY, USA) using a spin rate of 3000 rpm for 10 s. The coated film was subsequently heated to 80°C in an oven for 20 min to remove the excess solvent. During the procedure, the alkoxide film was partially hydrolyzed by ambient moisture to form amorphous vanadium oxides. Polycrystalline VO_2_ films were formed upon heating at 550°C for 30 min in a furnace under an Ar atmosphere via chemical vapor deposition. Subsequently, the VO_2_ film was slowly cooled to 23°C for use.

### EFUEM instrumentation

A 200-kV TEM (JEM-2100, JEOL, Tokyo, Japan) was modified to host two ports for optical access (one leading to the photocathode and the other to the specimen), each comprising an optical window and a mirror assembly. In addition, a weak, extra condenser lens (C0 lens, JEOL) was integrated between the electron gun and conventional condenser lens system to control the coherence and throughput of the photoelectron packets. A flat Ta cathode with a diameter of 840 μm was used (*14*, *62*). The gap between the surface of the photocathode and the exit aperture of the Wehnelt cup was set at 550 μm, and the electric field around the aperture was tuned to the highest Wehnelt bias (≈230 V), with a C0 lens potential of 23 V. A Yb-based amplifier (s-Pulse HP, Amplitude Systèmes, Pessac, France) was used to generate ultrashort pulses at 1030 nm with durations of 550 fs at 50 kHz. The frequency of one part of the femtosecond-laser output was doubled to 515 nm for the excitation of the specimen. The frequency of the other part was quadrupled to 257 nm and directed to the photocathode to generate electron probe pulses. The Δ*t* between the optical pump and probe electron pulses was tunable using an optical delay line via a motorized translational stage. The resulting time-framed micrographs, diffractograms, and EEL spectra were captured using a charge-coupled device camera (US4000, Gatan, Pleasanton, CA, USA) attached to the end of a postcolumn imaging filter (GIF Quantum SE, Gatan).

For EFUEM, the slit of the imaging filter was introduced at the central energy (Δ*E* = 0 eV) of the photoelectron bunch in a linear chirp regime. Among the gated electrons, those undergoing PINEM contributed to the micrographs, with a depletion of electron counts at Δ*t* = 0 and incremental changes a few picoseconds before and after Δ*t* = 0. This is revealed by the temporal evolution along Δ*E* = 0 eV shown in Fig. 2A. To selectively detect the structural dynamics of the NPs, the intensity modulation around the NP of interest by PINEM was extracted and applied to the original diffraction intensity profiles for normalization (fig. S13).

### Electron beam simulation

The generation of photoelectrons and their time-dependent distribution were simulated using a particle tracing module in COMSOL Multiphysics (COMSOL, Stockholm, Sweden), which is a finite element solver, as described in (*16*). The model studied in Fig. 2D comprises the Wehnelt cylinder, an accelerator, a drift tube, and apertures that reproduce the configurations of EFUEM. The photoelectron packet containing 3000 electrons per pulse at Δ*t* = 0 was initially released at the surface of the Ta cathode in a region with a diameter of approximately 100 μm with a Gaussian distribution, as photoemission is valid only at the center of the cathode. Photoemission followed Lambertian angular distributions, and the bias potential on the Wehnelt cylinder was 200 V.
