# Supplementary material for: Femtosecond-resolved imaging of a single-particle phase transition in energy-filtered ultrafast electron microscopy
Source: Sci Adv. 2023 Jan 27;9(4):eadd5375. doi: 10.1126/sciadv.add5375 (PMC9882981; doi:10.1126/sciadv.add5375)
Supplement: Supplementary file 1 — Figs. S1 to S13 [file sciadv.add5375_sm.pdf]

Science Advances
AAAS

Supplementary Materials for

Femtosecond-resolved imaging of a single-particle phase transition in energy-filtered ultrafast electron microscopy

Ye-Jin Kim *et al.*

Corresponding author: Oh-Hoon Kwon, ohkwon@unist.ac.kr

Sci. Adv. **9**, eadd5375 (2023)
DOI: 10.1126/sciadv.add5375

The PDF file includes:

Figs. S1 to S13
Legends for movies S1 to S4

Other Supplementary Material for this manuscript includes the following:

Movies S1 to S4

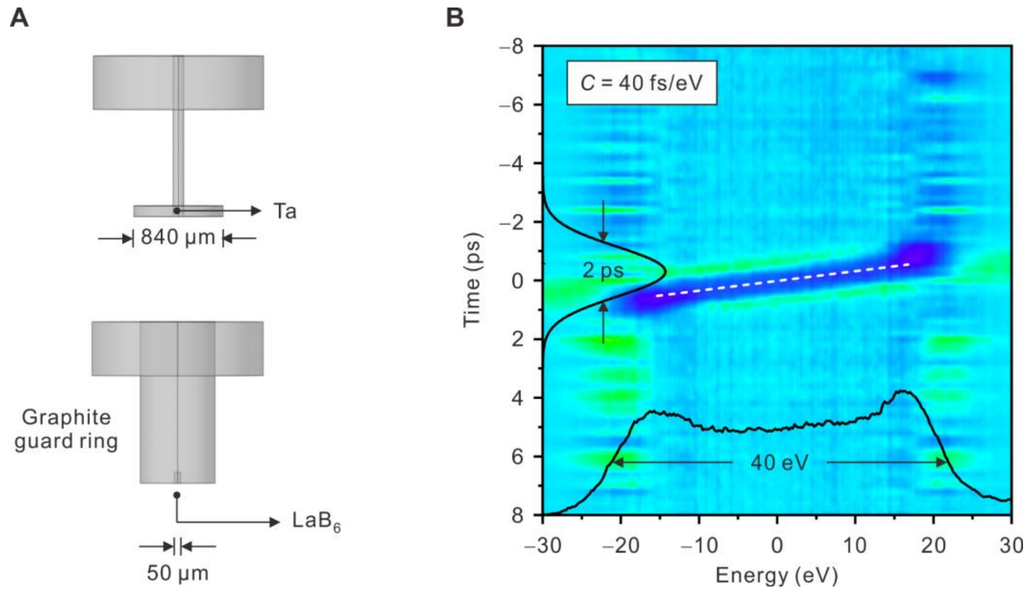

Fig. S1. Different photocathode geometries and chirps of photoelectrons. (**A**) Geometries of (top) a Ta cathode with a tip diameter of 840 μm and (bottom) an LaB_6 guard ring cathode surrounded by a graphite mantle with a tip size of 50 μm. (**B**) Energy-time (*E*–*t*) phase-space map of photoelectrons obtained using photon-induced near-field electron microscopy (PINEM) with the guard ring cathode. The chirp coefficient (*C*) is as low as 40 fs/eV, with a temporal resolution of approximately 2 ps.

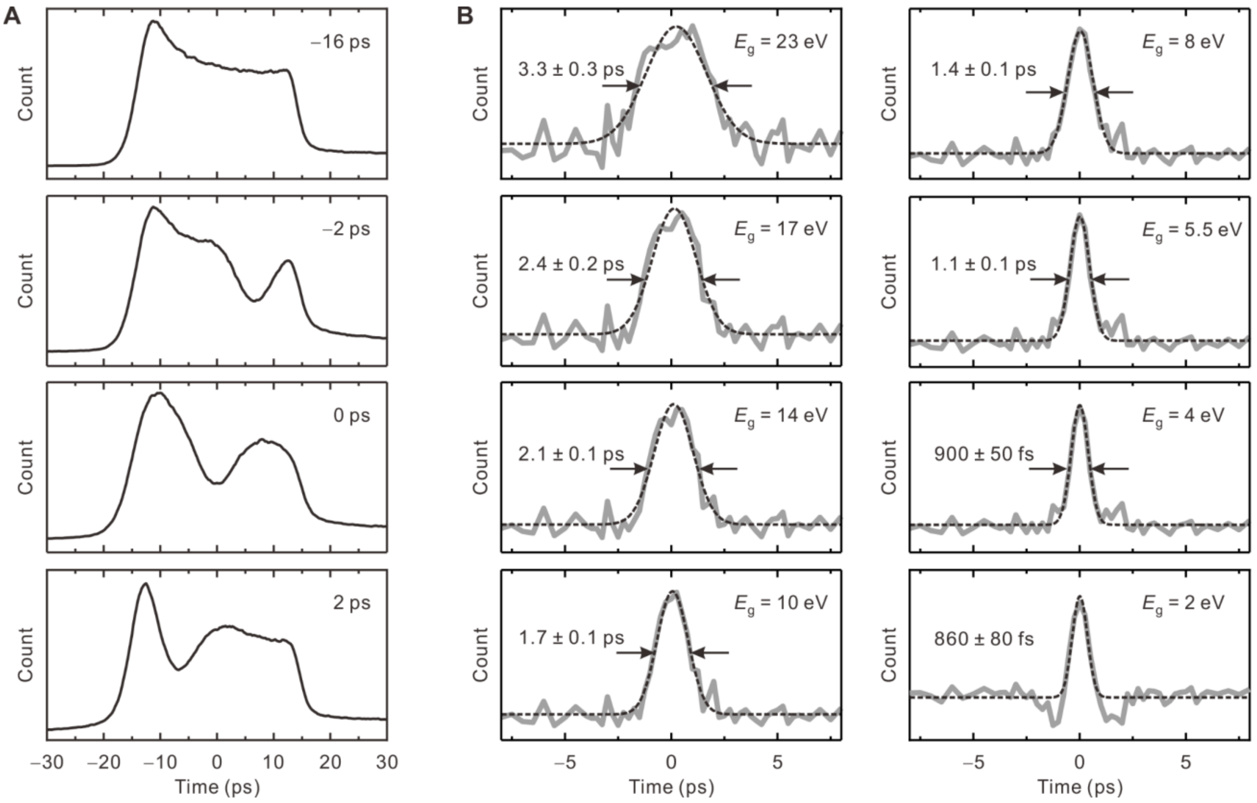

Fig. S2. Estimated temporal resolution obtained with energy gating. (**A**) PINEM spectra with intrinsic energy spread E_i = 30 eV at different Δt of –16, –2, 0, and 2 ps. (**B**) Temporal widths of gated photoelectron packets. Temporal resolutions (full-width at half-maximum of the envelope), which were estimated using the difference spectra referenced to Δt = –16 ps by selecting the slit width of the energy filter E_g, are also shown (refer to the plot shown in **Fig. 2f**).

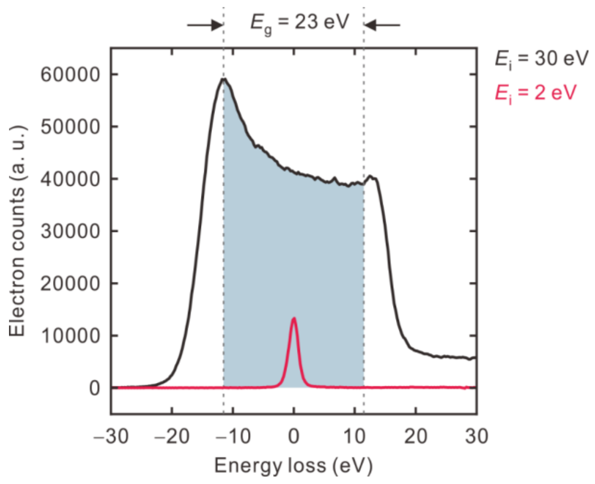

Fig. S3. Energy profiles of the photoelectron pulses with different E_i. At $t_i \approx t_g \approx 3$ ps, the photoelectron pulse of $E_i = 30$ eV gated with the slit width of 23 eV yields 36 times larger total electron count that the case of $E_i = 2$ eV.

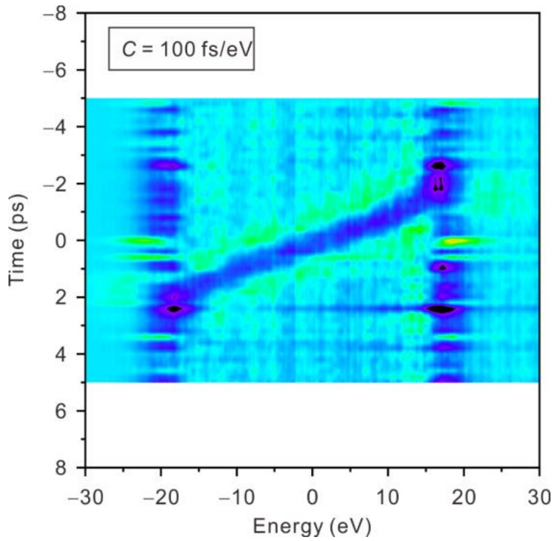

Fig. S4. Estimated temporal resolution obtained with energy gating. (*E*–*t*) phase-space map of photoelectrons obtained using PINEM with the Ta cathode for time-resolved imaging in **Fig. 4** and **5**. *C* is 100 fs/eV, with a temporal resolution and energy width of approximately 5.4 ps and 35 eV, respectively.

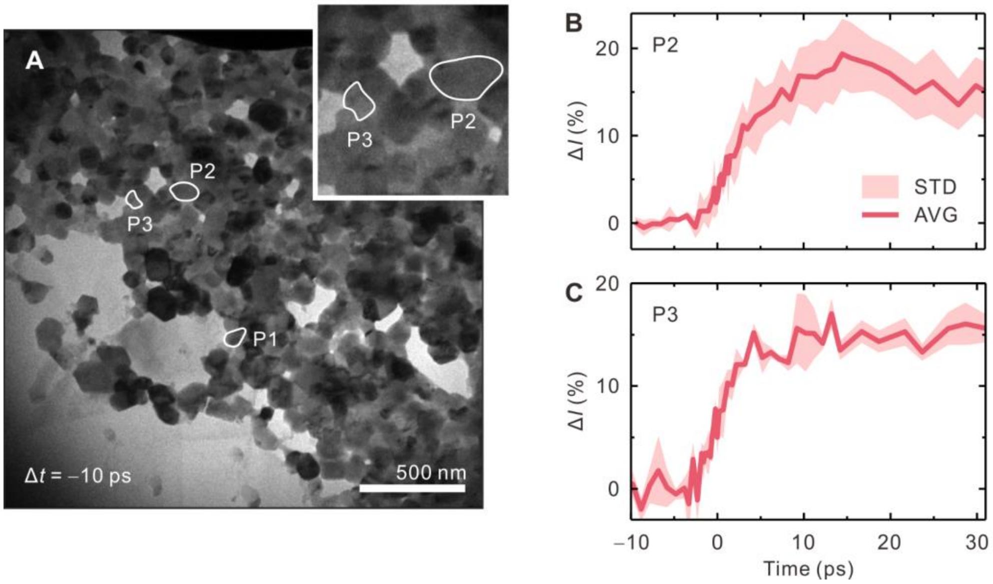

Fig. S5. Particles exhibiting instant phase transitions upon photoexcitation. (**A**) Bright-field image of VO_2 nanoparticles (NPs) captured with photoelectrons prior to photoexcitation (Δt = –10 ps). Particles P2 and P3 indicated inside the panel, along with P1, undergo ultrafast insulator-to-metal transitions (IMTs) upon photoexcitation. (**B** and **C**) Time-resolved intensity profiles of P2 and P3, respectively, obtained without energy filtering, *i.e.*, E_i = 35 eV, at F = 18 mJ/cm^2.

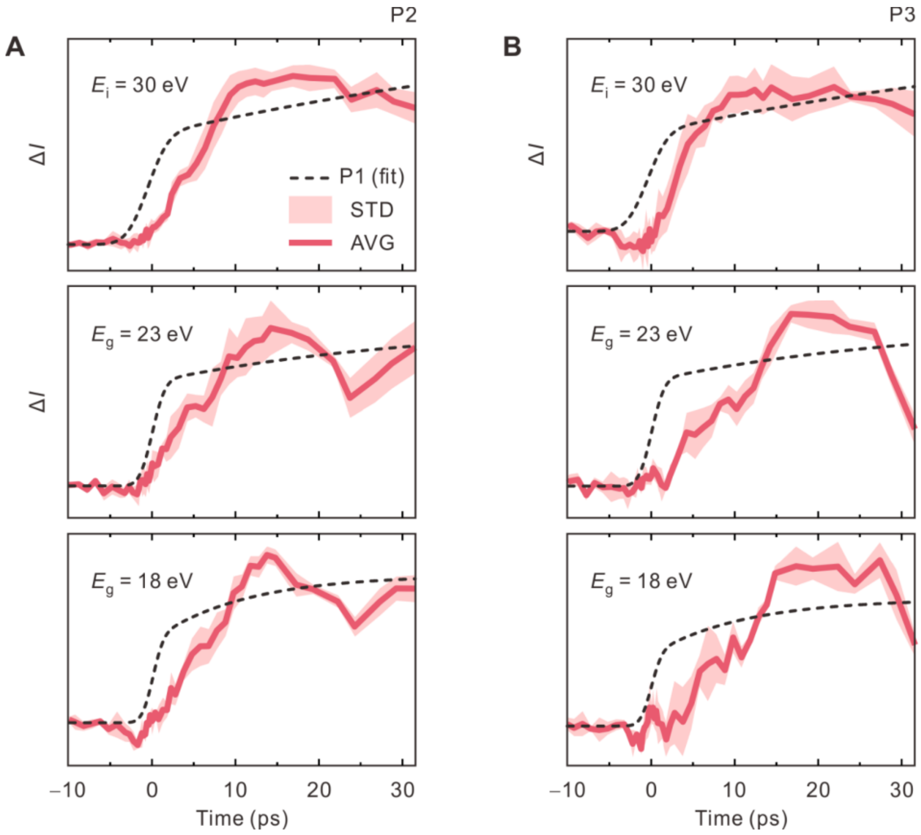

Fig. S6. Resolving the ultrafast IMT and slowly emerging intermediate state using gated photoelectrons. (**A** and **B**) Time-resolved intensity profiles of P4 and P5, respectively, obtained at F = 18 mJ/cm^2 captured using a photoelectron energy of 30 eV and E_g values of 23 and 18 eV. For comparison, fitted lines representing the changes in intensity of P1 obtained using different gating energies are also shown.

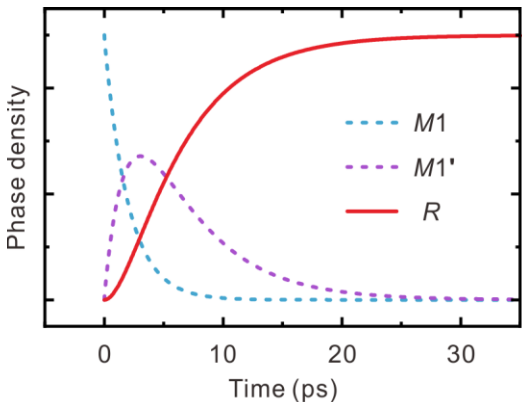

Fig. S7. Time-dependent populations of *M*1, *M*1', and *R* phases upon photoexcitation. Kinetic traces are modeled based on a consecutive two-step IMT ($M1 \rightarrow M1' \rightarrow R$); $d_{M1}(t) = d_{M1}(0)\exp(-k_1t)$, $d_{M1'}(t) = k_1d_{M1}(0)/(k_2 - k_1)[\exp(-k_1t) - \exp(-k_2t)]$, and $d_R(t) = d_{M1}(0)\{1 + [1/(k_1 - k_2)][k_2\exp(-k_1t) - k_1\exp(-k_2t)]\}$, where $d_{M1}(0)$, k_1, and k_2 represent the initial density of the photoexcited *M*1 phase and the rate constants of the first and second steps in the consecutive IMT, respectively. The k_1 and k_2 values used in the plot are 8.5×10^{11} and 2.2×10^{11} s^{-1}, respectively, which are based on the fit of P4 shown in **Fig. 5b**. Due to the consecutive nature of the phase transition, the induction period of a few picoseconds is observed prior to the formation of the final phase *R*.

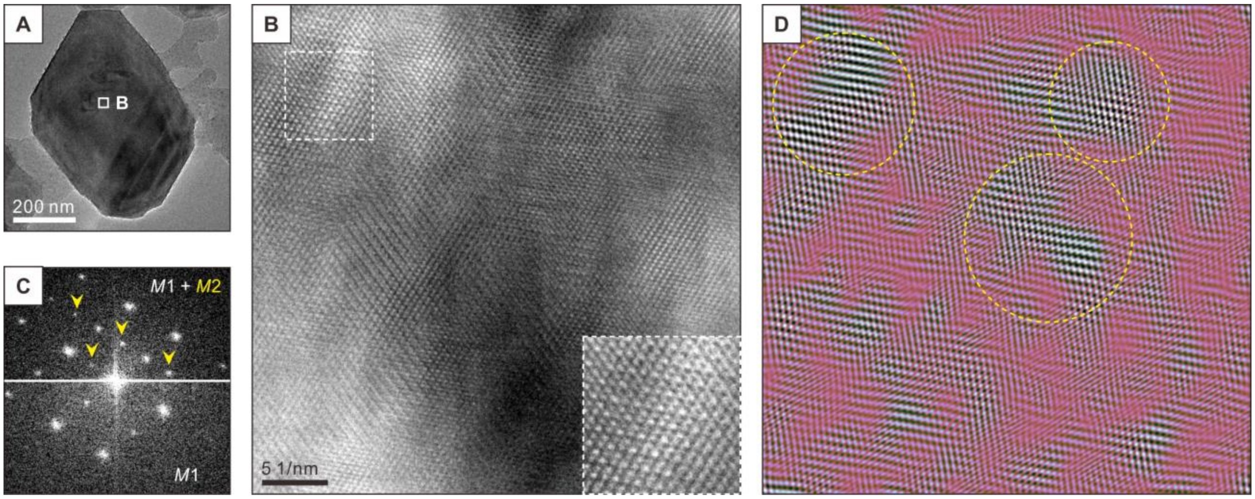

Fig. S8. Characterization of the steady-state *M*2 phase at room temperature. (**A**) TEM image of a VO_2 nanoparticle of interest at room temperature without photoexcitation. (**B**) High-resolution TEM image of the squared region in panel **A**. Inset is an enlarged view of the marked region where the *M*1 and *M*2 phases coexist. (**C**) (Upper) fast-Fourier transform (FFT) of the image in panel **B** which consists of both the *M*1 and *M*2 phases and (lower) FFT of another region with only the *M*1 phase. (**D**) Inverse FFT of the *M*2 reflections in panel **C**. High-contrast areas as highlighted with circles denote the *M*2-phased regions.

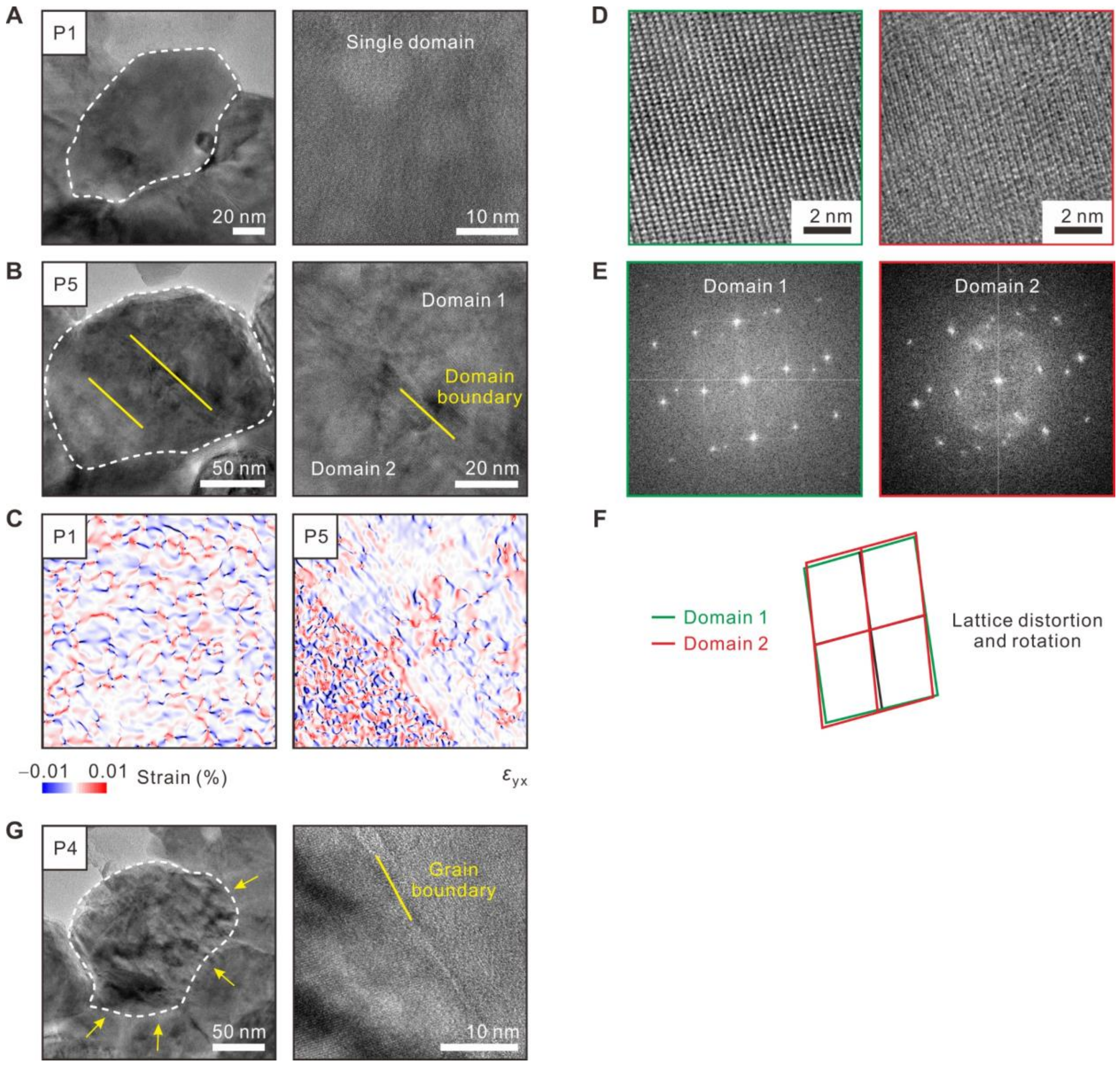

Fig. S9. Sources of particle strain. (**A** and **B**) Low- (left) and high- (right) magnification bright-field transmission electron microscopy (TEM) images of P1 and P5, respectively. Only a single domain occupies P1, resulting in the lowest strain, whereas two different crystalline domains (1 and 2) and a domain boundary are observed in P5, inducing a relatively high strain. (**C**) Strain map of the two regions in the right-hand panels shown in (**A**) and (**B**). (**D**) Lattice images of domains 1 and 2 in P5. (**E**) Corresponding fast Fourier transforms of the panels shown in (**D**). (**F**) Crystal symmetries of the two domains shown in panel (**E**). The lattice structures in domains 1 and 2 are distorted relative to each other. (**G**) Bright-field TEM images of P4. The larger size of the particle compared to that of P1 and the presence of the grain boundary likely result in higher strain.

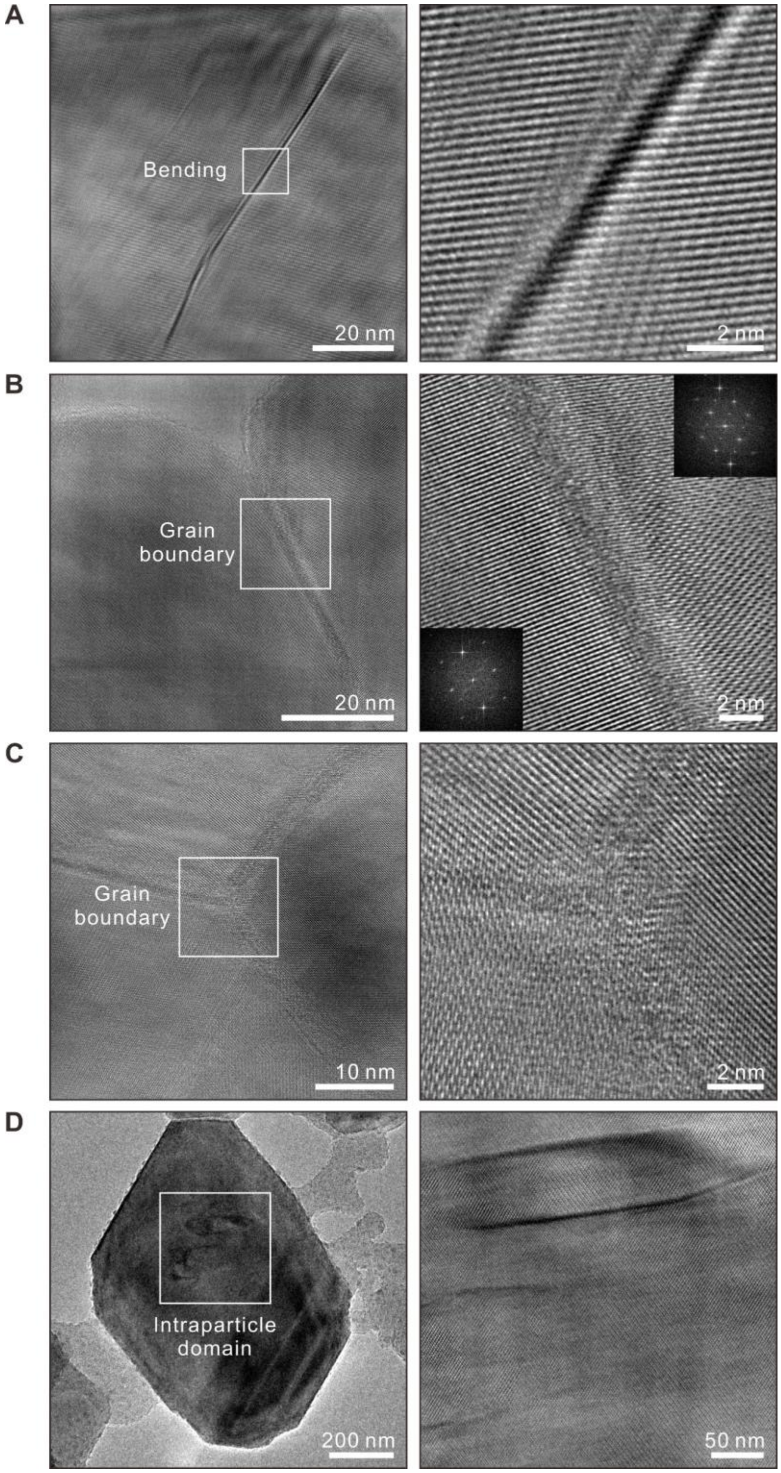

Fig. S10. Lattice structures of VO_2 NPs formed on the graphene substrate via chemical vapor deposition. Most of the NPs formed are single-crystalline with bending, grain boundary, and intraparticle domains, which are the main causes of local strain.

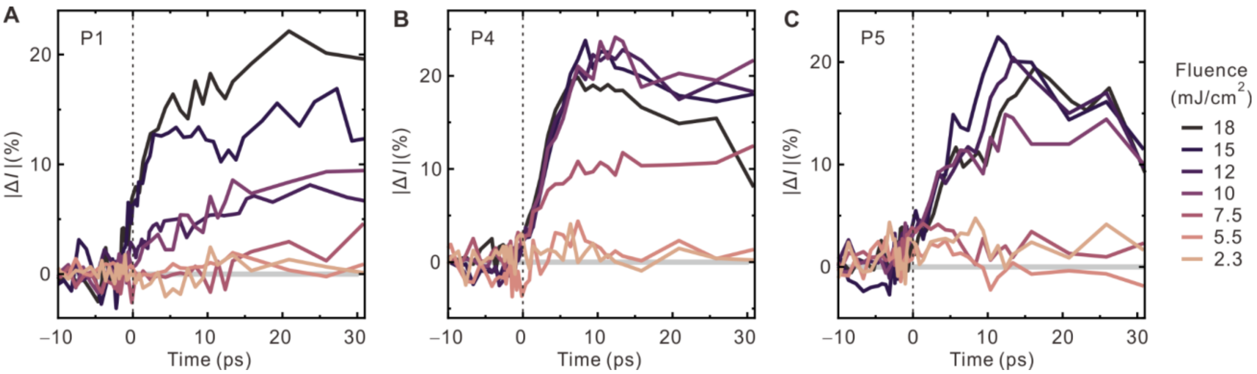

Fig. S11. **Fluence dependence of the phase transition.** (**A–C**) Time-resolved intensity profiles of P1, P4, and P5, respectively, at different excitation fluences, which are indicated on the right-hand side of the panels. If the delay in panels **B** and **C** originated from a thermal IMT, then the induction time would have been reduced with the excitation fluence because of the shift of the IMT mechanism from the slower thermal process to the ultrafast light-induced one. However, the delays in P4 and P5 are maintained in similar timescales (~1 ps) even at higher fluences, supporting that the delayed response is rather a signature of the emergence of the transient *M*2 phase than the thermally activated process.

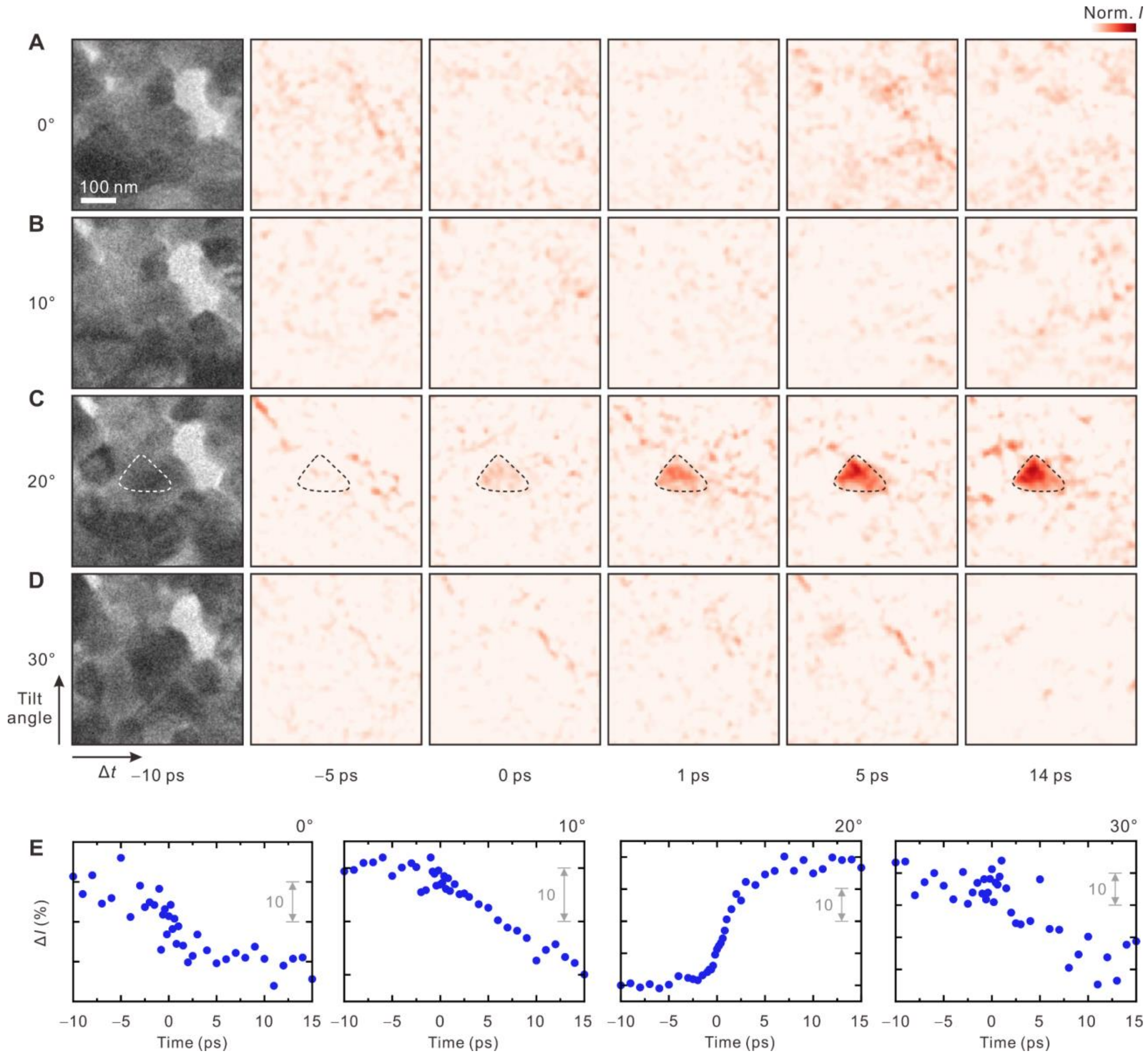

Fig. S12. Probe angle dependence of the visualization of the VO_2 phase transition. (**A-D**) False-color time-resolved images with different specimen tilt angles of 0°, 10°, 20°, or 30°, with F set at 40 mJ/cm^2. When the surface normal of the VO_2 film is tilted by 20° from the probe beam axis, the phase transformation of the marked area shown in panel (**C**) is observed immediately at $\Delta t = 0$. (**E**) Time-resolved intensity profiles of the marked areas shown in panels (**A-D**).

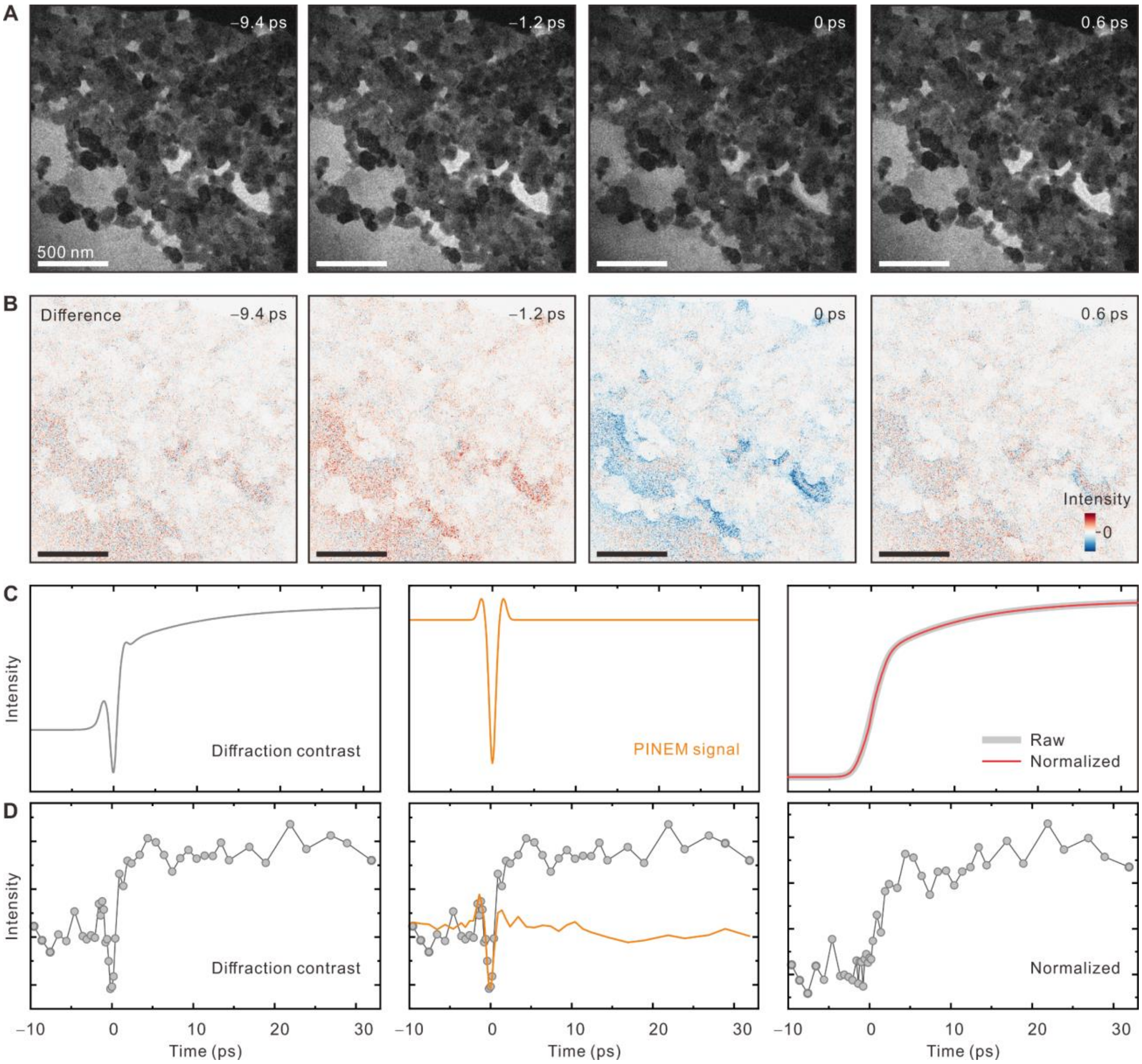

Fig. S13. Normalization of PINEM signal based on the time-framed images. (**A**) Time-framed images at different Δt. (**B**) Time-framed difference images referenced to $\Delta t = -10.4$ ps. (**C**) Simulated kinetic profiles of changes in the (Left) original diffraction contrast of NPs, (Middle) PINEM signal around the NPs, and (Right) PINEM-normalized kinetic profiles, which are identical. (**D**) Experimental kinetic profiles of changes in the (Left) original diffraction contrast of NPs, (Middle) PINEM signal around the NPs, and (Right) PINEM-normalized kinetic profile.

Movie S1.

***In-situ* imaging of phase transitions of VO_2 NPs.** *In-situ* heating and cooling of VO_2 NPs on a graphene/Si_3N_4 substrate. The temperatures of the corresponding images are indicated in the movie.

Movie S2.

Time-resolved imaging of phase transition of P1. (Top) Bright- and wide-field images captured using photoelectrons with ΔE = 35 eV at F = 18 mJ/cm^2. The corresponding time delays (Δt) are shown in the movie. (Bottom left) Magnified view of the marked region in the movie above. The periphery of P1 is outlined with a dotted line. (Bottom right) False-color image series of difference images referenced to Δt = –10 ps. Red indicates higher electron counts.

Movie S3.

Time-resolved imaging of phase transitions of P4 and P5. (Top) Bright- and wide-field images captured using photoelectrons with ΔE = 35 eV at F = 18 mJ/cm^2. The corresponding Δt are shown in the movie. (Bottom left) Magnified view of the marked region in the movie above. The peripheries of P1, P4, and P5 are outlined with dotted lines. (Bottom right) False-color image series of difference images referenced to Δt = –10 ps. Red indicates higher electron counts.

Movie S4.

Time-resolved imaging of the phase transition of a VO_2 NP ensemble. (Left) Bright- and wide-field images captured using photoelectrons with ΔE = 35 eV at F = 18 mJ/cm^2. The corresponding Δt are shown in the movie. (Right) False-color image series of difference images referenced to Δt = –10 ps. Purple indicates higher electron counts.
